# Autoimmune pathogenesis of gestational diabetes mellitus: the risk of progression to type 1 diabetes mellitus

**DOI:** 10.3389/fendo.2025.1663643

**Published:** 2025-10-07

**Authors:** Milena Skibińska, Marian Kacerovsky, Mariusz Grzesiak, Wioletta Izabela Wujcicka

**Affiliations:** ^1^ Department of Obstetrics and Gynecology, Polish Mother’s Memorial Hospital Research Institute, Lodz, Poland; ^2^ Department of Obstetrics and Gynecology, University Hospital Hradec Králové, Charles University, Hradec Králové, Czechia; ^3^ Biomedical Research Center, University Hospital Hradec Králové, Hradec Králové, Czechia; ^4^ Department of Perinatology, Obstetrics and Gynecology, Polish Mother’s Memorial Hospital Research Institute, Lodz, Poland; ^5^ Department of Gynecology and Obstetrics, Medical University of Lodz, Lodz, Poland; ^6^ Scientific Laboratory of the Department of Laboratory Diagnostics, Polish Mother’s Memorial Hospital Research Institute, Lodz, Poland

**Keywords:** GDM, T1DM, islet autoantibodies, beta-cell dysfunction, immune dysregulation

## Abstract

Gestational diabetes mellitus (GDM) is one of the most commonly diagnosed metabolic disorders in pregnancy, affecting between 5% and 20% of patients worldwide, depending on the diagnostic criteria and population. Although GDM pathogenesis is predominantly based on insulin resistance mechanisms resulting from the influence of pregnancy hormones, an increasing number of studies point to a significant role of immunological factors in the process of GDM development. In some GDM patients, autoantibodies targeting pancreatic beta cells are detected. Consequently, autoimmune processes may constitute an important element of GDM etiology, particularly in cases where GDM is a transitive condition leading to type 1 diabetes mellitus (T1DM) after the pregnancy. Disorders causing the destruction of beta cells within the pancreas precipitate permanent hyperglycemia in patients with autoimmune GDM (gestational diabetes mellitus with autoantibodies). characteristics. Genetic factors also play a significant role in this process, including single-nucleotide polymorphisms associated with the tissue compatibility system, such as HLA, CTLA-4, PTPN22 and IL2RA, which cause predisposition to T1DM. The following article discusses the current state of knowledge and presents GDM pathogenesis from the standpoint of immune mechanisms capable of affecting the development of this condition. It discusses potential markers that may help identify GDM patients at risk of progressing to permanent diabetes mellitus as well as possible diagnostic and therapeutic strategies based on the latest findings.

## Introduction

According to the 10^th^ edition of the IDF Diabetes Atlas from 2021, the global standardized prevalence of gestational diabetes mellitus (GDM) was 14.0% (1). GDM is associated with complications in both the mother and the infant. As Murray et al. indicate, GDM increases the risk of gestational hypertension, preeclampsia, as well as potential need for a caesarean delivery. The mother has also a significantly higher risk of developing type 2 diabetes mellitus at a later age, and the patients with autoimmune factors – also type 1 diabetes mellitus (T1DM). Untreated or inappropriately controlled hyperglycemia may result in excessive fetal growth (macrosomia), which carries the risk of complications during delivery, such as shoulder dystocia, perinatal injuries or asphyxia. Neonates of GDM mothers are also more prone to hyperglycemia, and other disorders like hypokalemia, hypomagnesemia or neonatal jaundice. Additionally, long-term consequences of GDM include an increased risk of obesity, insulin resistance and type 2 diabetes mellitus (T2DM) in children who were exposed to maternal hyperglycemia *in utero* (2). GDM also affects the child’s neuropsychological development – some studies indicate its correlation with attention disorders and a higher risk of attention deficit hyperactivity disorder (ADHD) (3).

The scale of potential complications largely depends on the time of the diagnosis, treatment efficacy and the level of glycemic control during pregnancy. The International Association of the Diabetes and Pregnancy Study Groups (IADPSG) currently recommend a universal, single-stage screening approach with a 2-hour oral glucose tolerance test (OGTT) using 75 g of glucose administered between 24 and 28 weeks of gestation, which involves rigorous diagnostic criteria to detect GDM. According to the recommendations based on the Hyperglycemia and Adverse Pregnancy Outcome (HAPO) study, GDM is diagnosed when the fasting blood sugar level is ≥ 5.1 mmol/L or during the OGTT ≥ 10 mmol/L at 1 hour or ≥ 8.5 mmol/L at 2 hours after glucose administration (4). Blood sugar levels usually return to normal shortly after delivery. However, women with a history of GDM are more likely to later develop T2DM, cardiovascular diseases and metabolic syndrome, compared to women with normal glucose tolerance (5).

The basic mechanisms of GDM development include an increase in insulin resistance triggered by hormones produced during gestation, like human placental lactogen (hPL), estrogens and cortisol. The presence of antibodies against pancreatic beta cells in some patients with GDM also suggests a potential autoimmune cause of this condition and possibly an initial stage of autoimmune destruction of beta cells. These changes may result in T1DM following the pregnancy (6).

The aim of this paper is to summarize the currently available evidence of the involvement of autoimmune mechanisms in GDM pathogenesis and their potential connection to T1DM development after gestation. The article presents new perspectives and conclusions based on recent studies, including an analysis of the immune processes’ significance in diagnostics, monitoring and clinical management of GDM patients.

## Gestational diabetes and the risk of type 1 diabetes mellitus

**Table 1** (Supplementary material) shows the current diagnostic criteria for diabetes according to the World Health Organization (WHO) – fasting glycemia ≥ 7.0 mmol/L, confirmed twice on different days, glycemia 2 hours after glucose administration in OGTT ≥ 11.1 mmol/L, random blood glucose level ≥ 11.1 mmol/L accompanied by typical symptoms of hyperglycemia and glycated hemoglobin (HbA1c) ≥ 6.5% (7). To confirm a diagnosis of T1DM in cases where at least one diagnostic criterion for DM is met, it is necessary to identify the presence of autoantibodies (anti-islet antibodies, antibodies to glutamic acid decarboxylase [GAA or GAD], protein tyrosine phosphatase 2 [IA2 or ICA512], endogenous insulin [IAA], or anti-zinc transporter 8 antibodies [ZnT8A]) and/or a decreased concentration of the C-peptide, which reflects endogenous insulin levels (1).

**Table 1:** Diagnostic criteria for diabetes mellitus according to the WHO (7).

Test	Fasting blood sugar level	Glycemia 2 h into OGTT	Random glycemia	HbA1c
Diagnosis of DM if ≥ 1 is/are met	≥7.0 mmol/L twice (on 2 separate days)	≥11.1 mmol/L	≥11.1 mmol/L with typical symptoms of hyperglycemia (polydipsia, polyuria, weakness, diabetic ketoacidosis)	≥6.5 % (48 mmol/mol)

T1DM is caused by the destruction of pancreatic beta cells through an autoimmune (in most cases) or undetermined process, which usually leads to an absolute insulin deficiency. If there is a genetic predisposition, once the triggering agent occurs, an autoimmune reaction develops and the pancreatic islets become inflamed (insulitis) (8). The triggering agent causes autoantigen expression. The antigens released from the beta cells are processed in macrophages and then in communication with class II Human Leukocyte Antigen (HLA) presented to CD4 T lymphocytes. The activated T lymphocytes trigger a cellular and humoral response and they release cytokines: interleukin-2 (IL-2) and interferon gamma (IFNG or IFN-γ). Cytokines activate cytotoxic CD8 T lymphocytes and NK cells, while the activated B lymphocytes produce autoantibodies. IFN-γ from T lymphocytes stimulates macrophages, which then release free radicals and nitric oxide, substances that are toxic to the beta cells. By this process, the destruction of beta cells progresses until their antigens are depleted (9).

The development of autoimmune-based diabetes involves antibodies which may appear many months or even years before the patient shows any symptoms (10). During that period, beta cells gradually lose their secretion potential resulting in manifest diabetes caused by an absolute insulin deficiency. The disease course depends on the rate of beta-cell loss. Initially, first-phase insulin release atrophy occurs, followed by prediabetes and finally manifest diabetes mellitus. Typically, the condition occurs in children and adolescents as well as people below the age of 30. Once the disease manifests, the destruction of beta cells continues for some time. The disappearance of C-peptide (insulin secretion marker) from the serum signifies complete beta-cell degradation (11). A role in initiating the autoimmune process is attributed to viruses: mumps virus (MuV), coxsackie B virus, cytomegalovirus (CMV), retroviruses (i.e. HIV), rubella virus (RuV), Epstein–Barr virus (EBV), hepatitis A virus (HAV), poliovirus, and influenza viruses. Viruses may damage beta cells directly or trigger an autoimmune response (8). Food proteins from cow’s milk (12) and cereals may have a similar effect (13).

American Diabetes Association recommends regarding any slowly progressing autoimmune-related diabetes with an adult-age onset as T1DM due to the common aetiology involving the destruction of pancreatic islet cells, even if the process occurs at a slower rate (14). The course of T1DM in young adults is not always dynamic, the symptoms may increase slowly, sometimes over a few months. The full clinical picture emerges in the patients’ 40s and 50s. It may cause such patients to be misdiagnosed with T2DM and put on oral medication, which may be initially effective due to the preserved residual insulin secretion capacity. In practical terms, the distinction between this type of disease and T2DM is crucial, as the majority of patients with the diagnosis of autoimmune diabetes requires insulin therapy implementation within a short period after diagnosis due to the progressive loss of beta-cell function. The long-term course of seemingly mild hyperglycemia, involving even a temporary regression of biochemical disturbances, promotes the development of chronic complications (15).

During pregnancy, a rise in the concentration of hormones with an insulin-antagonistic effect leads to insulin resistance and increased insulin demand, which may constitute a triggering factor resulting in earlier disease manifestation. It is also a period of intense immune changes which may accelerate the process of latent autoimmunity. In women already suffering from subclinical T1DM, gestation may precipitate beta-cell destruction leading to manifest diabetes after delivery (16). **Table 2** shows a summary of environmental factors which may potentially contribute to the autoimmune process activation within the pancreas.

**Table 2:** The influence of environmental factors on the risk of GDM progression to T1DM.

Environmental factor	Mechanism of action	T1DM	Source
Viral infections (enteroviruses, Coxsackie B, rotaviruses)	Molecular mimicry, autoimmune reaction activation against beta cells	High in genetically predisposed patients	(17, 18)
Hyperglycemia and inflammation in pregnancy	A rise in IL-6, IL-17, TNF-alfa leading to beta-cell destruction	Medium to high	(19, 37)
Diet rich in trans fats and simple sugars	Oxidative stress, exacerbation of the inflammatory process, insulin resistance	Medium	(20, 21),
Vitamin D deficiency	Weakened immune regulation, increased susceptibility to autoimmunity	Medium to high	(22, 23)
Environmental pollutants (BPA, PCBs, PM2.5)	Intensification of oxidative stress and immune responses	Medium	(24)

GDM, gestational diabetes mellitus; T1DM, type 1 diabetes mellitus; IL-6 –interleukin 6, IL-17, interleukin 17; TNF-alfa, tumor necrosis factor alpha; BPA, bisphenol A; PCB, polychlorinated biphenyls; PM2.5, particulate matter 2.5.

## Autoantibodies in gestational diabetes

In newly diagnosed type 1 diabetes, autoantibodies are present in 80–90% of patients (25). In a recent population-based study, Lomeli et al. reported that diabetes-associated autoantibodies were present in 13.6% of patients with T2DM (26). In adults with impaired glucose tolerance (IGT), autoantibodies are detected in about 4% with no significant prognostic relevance for the development of diabetes (27). Research findings indicate that approximately 5-10% of women diagnosed with GDM test positive for autoantibodies, which suggests an autoimmune component in beta-cell dysfunction. In GDM patients with autoantibodies, the risk of progression to T1DM within a year may be as high as 50% (28). The autoimmune GDM therefore occupies an intermediate position between T2DM and T1DM with a risk of progression to autoimmune diabetes that is significantly higher than in T2D and IGT, but lower than in overt T1DM.

Autoantibodies associated with diabetes:

- GADA (glutamic acid decarboxylase antibodies) present in 5-10% of women GDM and associated with a higher risk of progression to T1DM postpartum (29),
- less common ICA (islet cell antibodies) associated with autoimmune beta-cell damage (30),
- IA-2A (insulinoma‐associated antigen‐2) (31, 32),
- IAA (insulin antibodies), present mainly in young patients, which suggests an early stage of autoimmunity (33) and
- ZnT8 autoantibodies (zinc transporter 8 antibodies) (34).

Islet cell antibodies (ICA) are detected using indirect immunofluorescence, while the presence of the remaining antibodies, such as GADA, IA-2A, IAA and ZnT8A, is determined with automated immunochemical techniques like the radioimmunoassay (RIA) or enzyme-linked immunosorbent assay (ELISA) (35). In the course of autoimmune process, or in fact once it is complete, following the destruction of beta cells within the islets of Langerhans, anti-islet antibodies disappear and in later stages of T1DM may be completely absent (36).

**Figure 1:**
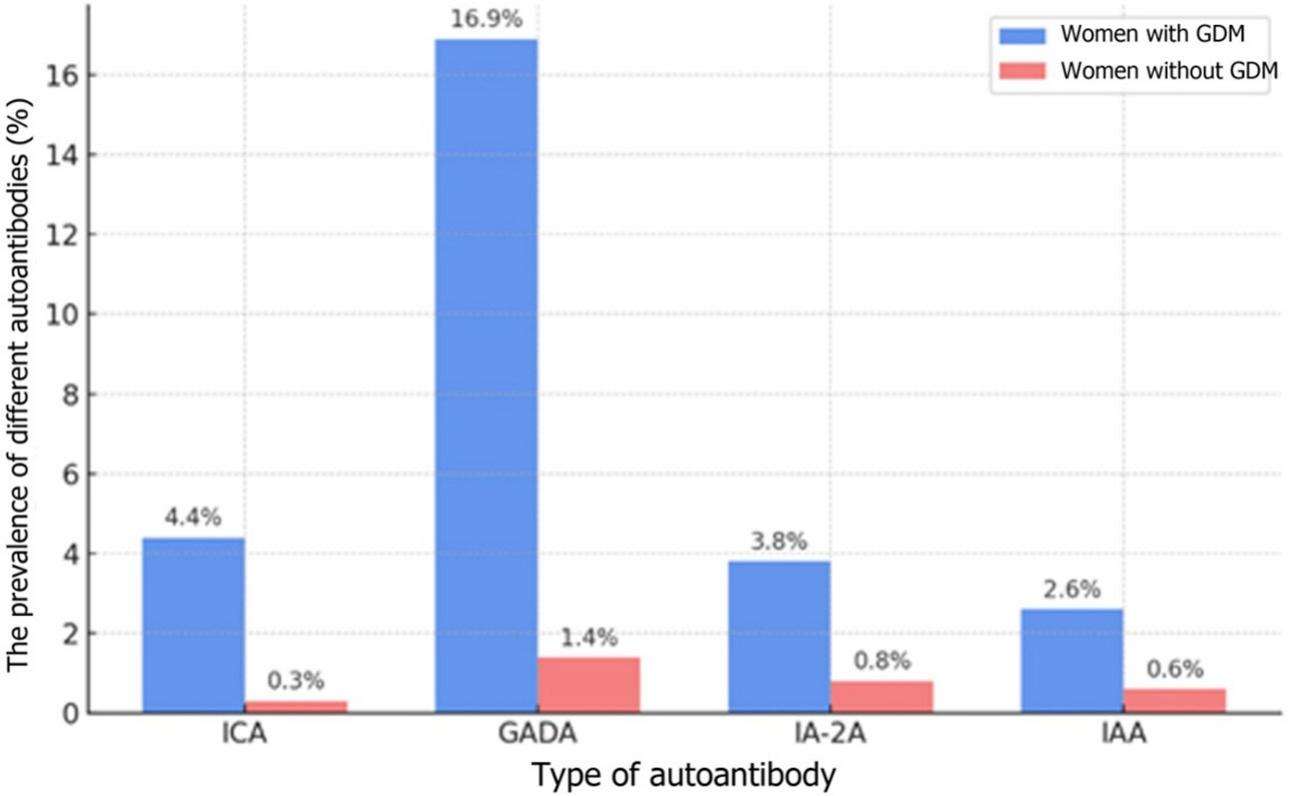
The prevalence of different autoantibodies in patients both with and without GDM (32) The types of autoantibodies: ICA-islet cell antibodies; GADA-glutamic acid decarboxylase antibodies, IA-2A - insulinoma-associated antigen; IAA - insulin autoantibodies; 12% refers to the overall prevalence of any autoantibodies, whereas the rates in the table originate from the same cohort but pertain to each individual marker.

In 2019, Incani et al. analyzed the prevalence of T1DM-related autoantibodies in women with GDM in various tests as well as their clinical implications. The study demonstrated that autoantibodies associated with T1DM occur in less than 10% of women with GDM. Consequently, the authors suggest that routine testing for these autoantibodies in GDM patients may be warranted only in cases where autoimmune etiology is suspected, since they tend to be rare and there are no significant differences between the course of GDM pregnancies with and without the presence of autoantibodies (28). In 2022, Beunen et al. again evaluated the presence of diabetes-related autoantibodies in GDM patients. The results showed a relatively low percentage of women with autoantibodies and revealed a link between these antibodies and an increased risk of developing glycemic disorders after the pregnancy (6). A recent study by Luiro et al. analyzed observational data obtained over a period of 23 years and showed that 12% of GDM patients tested positive for at least one autoantibody, compared to 2.3% in the control group. The presence of three different autoantibodies was associated with the highest risk of developing T1DM within 7 years after gestation. The findings suggest that monitoring GADA and ICA levels in early pregnancy may be crucial in identification of GDM women susceptible to T1DM (32). The prevalence of different autoantibodies in patients both with and without GDM is shown in **Figure 1**.

In the available studies, the prevalence of autoantibody detection in women with GDM is highly heterogeneous, ranging from 3% to 12%. These differences primarily result from varying diagnostic criteria for GDM, the laboratory methods used (RIA vs. ELISA, different commercial kits), as well as population diversity. In Scandinavian studies, the proportion of women with GDM who had autoantibodies exceeded 10% (32), whereas in Asian populations, reported values were more often in the range of 3–6% (28), which may suggest the influence of genetic and environmental factors. Such heterogeneity constrains the generalizability of the findings and highlights an important gap in the current evidence base.

## Autoimmune mechanisms in gestational diabetes

The autoimmune mechanisms potentially associated with GDM include T cell dysfunction, overproduction of proinflammatory cytokines, and genetic predispositions like HLA system gene variants.

### T cell dysfunction in GDM

Human immune system maintains a dynamic balance between effector T cells (mainly Th1 and Th17) and regulatory T cells (Tregs or Treg cells), which allows for an effective immune response without excessive inflammation. Normal pregnancy is characterized by Treg expansion and Th17 suppression, which ensures fetal tolerance (37). By contrast, GDM with autoantibodies is characterized by Treg dysfunction and a persistently overactive Th17 response, which may result in chronic inflammation, damage to pancreatic beta cells and impaired glucose homeostasis (38). A study conducted by Sifnaios et al. found that in the 3^rd^ trimester of gestation, women with GDM showed a higher percentage of Th2, Th17 and Treg cells, compared to healthy pregnant females. Also, the T cell profile remained altered for at least 6 months after delivery, which suggests long-term immune disorders (39). Th17 helper cells are crucial mediator cells in pro-inflammatory response through their release of interleukin 17 (IL-17), which stimulates the activation of neutrophils and boosts inflammation (37). In women with GDM, overactive Th17 cells may contribute to beta-cell damage and the progress of insulin resistance. Conversely, the activity of Treg cells, whose function is to suppress excessive immune responses, appears to be lower in GDM patients, which leads to rampant inflammation (19).

Hyperglycemia plays a key role in disruptions to the Th17 and Treg cell balance. Research suggests that a high concentration of glucose may promote the differentiation of naive T cells (Th0 cells) into Th17 while suppressing the development of Treg cell population. Additionally, cytokines like IL-6 and TGF-beta regulate this process – IL-6 facilitates Th17 differentiation, whereas TGF-beta supports Treg development. In women with GDM, an increased level of IL-6 promotes a predominance of TH-17 over Treg, which further exacerbates the inflammatory process (40). According to research, GDM women show an altered expression of immune checkpoint receptors, such as PD-1 and CTLA-4. Notably, decreased CTLA-4 expression among T cells in patients with GDM may lead to exaggerated T cell activation and prolonged inflammation. Also, an increased PD-1 expression among T cells suggests a dysregulation of immune tolerance mechanisms in GDM. These changes may contribute to the immune imbalance and chronic inflammation observed in GDM patients (41, 42). T lymphocyte dysfunction in GDM may have far-reaching consequences which leads to the loss of tolerance to beta cells and the progression of autoimmunization. Chronic inflammation leads to increased insulin resistance and damage to beta cells, which further deteriorates glycemic control in women with GDM (19). Furthermore, it must be emphasized that children of GDM mothers are at a higher risk of developing autoimmune diseases, including allergies and atopic dermatitis (AD). It may result from prenatal exposure to an altered immune environment where over-activated Th17 cells and underactive Treg cells contribute to the development of chronic inflammation (40).

T cell dysfunction plays a significant role in GDM pathogenesis. Specifically, a lower number of Treg cells in the peripheral blood of GDM women may encourage the disease to develop. Modulating the balance between different T cell subpopulations, especially by regulating the levels of cytokines like IL-6 and TGF-beta may constitute a potential therapeutic strategy to improve glycemic control and reduce inflammation in GDM patients. Interventions aimed at reducing the inflammatory process and restoring Treg function may facilitate GDM treatment and complication prevention (43).

### Pro-inflammatory cytokines

One of the key mechanisms which exacerbate inflammation in GDM is overproduction of pro-inflammatory cytokines like interleukin-6 (IL-6), interleukin-17 (IL-17) and tumor necrosis factor alpha (TNF-alpha) (44). Not only do these cytokines intensify the inflammatory process, but they also play a significant role in the development of insulin resistance and pancreatic beta-cell dysfunction, which leads to abnormal regulation of blood glucose levels (45). IL-6 is one of the body’s main inflammatory mediators and plays a major role in immune response regulation. In GDM, IL-6 levels were found to be substantially elevated in pregnant women suffering from this disease, compared to healthy patients (46). IL-6 promotes insulin resistance through the activation of JAK-STAT signaling pathway and impacts adipocyte dysfunction, leading to excessive lipolysis and an increased level of free fatty acids (47). Similarly, as a key proinflammatory agent, TNF-alpha contributes to the progress of insulin resistance by suppressing insulin signaling and stimulating prolonged inflammation. Studies show an increased TNF-alpha concentration in GDM women, and its levels correlate with the severity of hyperglycemia and the degree of insulin resistance (42). Additionally, TNF-alpha may adversely affect beta-cell function by inducing oxidative stress and apoptosis, further impairing glycemic control (48). IL-17 produced mainly by Th17 cells is a strong inflammatory promoter and participates in the pathogenesis of many autoimmune diseases, including GDM. High IL-17 levels in GDM patients indicate its role in boosting the autoimmune inflammatory response and beta-cell destruction (39). An increased level of IL-17 weakens the intestinal barrier and leads to a rise in the release of endotoxins, which further stimulate the immune system to produce substances promoting inflammation (49). Autoimmune processes in GDM are closely associated with abnormal levels of cytokines, including TNF-alpha, IL-6 and IL-1beta. IL-1beta is crucial in immune response regulation through the NLRP3 inflammasome activation and oxidative stress induction (50). GDM studies show that IL-1beta may directly affect beta-cell function impairment, resulting in their dysfunction and apoptosis (51). Research results suggest that women with GDM have lower concentrations of IL-1Ra (Interleukin-1 Receptor Antagonist), which may be associated with the postpartum development of T2DM (52). Moreover, chronic inflammation in GDM patients may lead to long-term health complications in both the mother and the child. Research shows that children of GDM mothers are more susceptible to insulin resistance and metabolic syndrome in later life (53). Furthermore, the inflammation may contribute to the development of preeclampsia in pregnancy and cardiovascular complications (54).

To sum up, pro-inflammatory cytokines, such as IL-6, TNF-alpha, IL-17 and IL-1beta, play a key role in the pathogenesis of GDM by stimulating the inflammatory process and leading to beta-cell function impairment. The overexpression of proinflammatory cytokines contributes to the development of insulin resistance and metabolic disorders, which may result in serious complications in both the mother and the child. Understanding molecular mechanisms associated with cytokine activity in GDM may help develop new therapeutic strategies aimed at controlling the inflammatory process and improving the prognosis in patients affected by this condition.

## Genetic predisposition- the role of HLA

Genetic predisposition plays a key role in the development of autoimmune diseases like GDM. An increasing number of studies indicates that some gene variants linked to the immune system correlate with an increased risk of developing this condition (55). In particular, genetic variations within the human leukocyte antigen (HLA) system may affect the immune response and autoimmune process development (56). Additionally, the HLA system – a key element in immune response regulation – shows numerous polymorphisms which may predispose to GDM (57). Many researchers point out specific SNPs within the HLA system, such as HLA-DR3 and HLA-DR4, which have been linked to T1DM (58). Their increased prevalence in women with GDM may indicate similar pathogenic mechanisms behind the two conditions (56). According to Lapolla et al., the existence of these variants may imply a common genetic basis for T1DM and GDM (59). Furthermore, other polymorphisms within the HLA complex, such as HLA-DQB1 and HLA-DRB1, have also been linked to increased susceptibility to metabolic disorders in pregnancy (57).

Genetic predisposition to GDM may be further potentiated by environmental factors, like metabolic stress in gestation, obesity or lifestyle. Analogous to genetic factors, metabolic stress may likewise trigger the activation of inflammatory pathways leading to a stronger immune response (59). Chronic inflammation associated with pregnancy may modulate the expression of genes responsible for the immune system regulation, which triggers an increase in the production of proinflammatory cytokines like IL-6 or TNF-alpha (60). Also, a significant factor is the pregnant woman’s diet. According to research, excessive ingestion of simple sugars and saturated fatty acids may exacerbate the immune response causing increased insulin resistance and GDM in genetically predisposed women (61).

Gene variants within the HLA system play a key role in GDM pathogenesis, which is confirmed by numerous studies. Gene interaction with the environmental factors, like metabolic stress or diet, may induce an autoimmune response leading to metabolic disorders in pregnancy.

## Other genetic factors

To date, selected single nucleotide polymorphisms in CTLA4 have been linked to the susceptibility to T1DM. Particularly, the +49A/G SNP in exon 1 of CTLA-4 was associated with an increased risk of developing T1DM. A study involving the population of Ethiopia showed that the G allele of that SNP is more common in T1DM patients than in the control group, which implies its role in the condition’s pathogenesis. However, a link between CTLA4 SNPs and GDM remains to be unequivocally confirmed. Further research is needed into the potential connection between CTLA4 variants and GDM development. Other studies also indicate a link between SNPs in PTPN22 and an increased risk of T1DM. Specifically, the rs2476601 (c.1858C>T) variant has been identified as a risk factor for T1DM in Caucasian populations. A 2023 study involving patients of Armenian descent showed that the T allele in rs2476601 occurs more frequently in individuals with T1DM than in the control group, which suggests its role in the pathogenesis of the disease (62). SNPs in INS (insulin) gene, in turn, have been linked to the regulation of insulin autoantigen expression which may increase the risk of autoimmune beta-cell destruction (63). Research shows that SNPs in IL2RA and IFIH1 genes are associated with the susceptibility to autoimmune diseases, including T1DM. The IL2RA (Interleukin-2 Receptor Alpha) gene codes the receptor for interleukin-2, which is crucial for the homeostasis between effector and regulator T cells. A 2015 meta-analysis revealed that five SNPs in IL2RA are significantly linked to a higher risk of T1DM. Notably, **r**s11594656, rs2104286 and rs41295061 showed the strongest correlation with susceptibility to T1DM (64). More recent studies also confirm a connection between rs1990760 in IFIH1 and the susceptibility to T1DM. A 2023 meta-analysis revealed that the A allele of this SNP increases the risk of autoimmune diseases, especially within the Caucasian population (65).

The autoimmune mechanisms behind GDM are complex and multifactorial. T cell dysfunction, overproduction of pro-inflammatory cytokines and genetic predispositions, such as HLA-DR3 and HLA-DR4 variants, are all crucial in the pathogenesis of this condition. Understanding these mechanisms is critical not only for diagnostic purposes, but also to develop effective strategies of treatment and prevention of GDM progression into permanent forms of autoimmune diabetes. Further research in this area may contribute to a better understanding of the interactions between the immune system and metabolism in gestation (66).

Among the genetic factors suspected to increase the risk of autoimmune GDM are HLA-DR3/DR4 alleles and polymorphisms in the CTLA4, PTPN22, and INS genes. However, it should be noted that estimates of effect size derive almost exclusively from studies on T1D. In the case of GDM, large association studies are lacking, and the available reports remain preliminary.

**Table 3** shows a summary of potential autoimmune mechanisms in gestational diabetes.

**Table 3:** Autoimmune mechanisms in gestational diabetes.

Mechanism		Description	Source
Autoantibodies	GADA (against glutamic acid decarboxylase)	Detected in some women with GDM, suggest a risk of developing T1DM.	(29, 32, 59)
	ICA (against pancreatic islet cells)	Less common in GDM patients	(30, 32, 59)
	IA-2A (against tyrosine phosphatase-like protein IA-2)	More common in GDM patients, indicate damage to beta cells.	(32, 59, 67)
	ZnT8A (against zinc transporter ZnT8)	Occur in T1DM, a suggested factor in GDM.	(34, 68)
Immune system cells	T cell dysfunction	Balance dysruption between Th1/Th17 and Treg cells leading to chronic inflammation	(19, 69)
	Decreased Treg cell activity	Weakened Treg cell function promotes uncontrolled inflammatory response	(19, 70–72)
	Increased Th17 cell activity	Increased Th17 activity and IL-17 production lead to beta-cell damage.	(39, 40, 69, 71)
Cytokines	Overproduction of proinflammatory cytokines	An increase in IL-6, IL-17 and TNF-alpha cytokine levels contributes to insulin resistance and beta-cell damage.	(44, 73)
	Increased IL-6 level	High IL-6 levels promote insulin resistance via the JAK/STAT pathway and changes to adipocyte cell metabolism.	(47, 74)
	Increased IL-17 level	IL-17 increases inflammation and damages the intestinal barrier by increasing endotoxin production.	(37, 69, 71)
	Increased IL-1beta concentration	Beta-cell impairment resulting in their dysfunction and apoptosis.	(51, 73, 75, 76)
Genetic factors	HLA-DR3, HLA-DR4 variants	Increased susceptibility to autoimmune forms of diabetes.	(57)
	SNPs in CTLA4 gene	T cell regulation and their tolerance capacity towards autoantigens. Linked to T1DM and other autoimmune diseases.	(62, 72)
	SNPs in PTPN22 gene	Increased autoreactive T cell activation, significant in autoimmune forms of GDM.	(62, 77, 78)
	SNPs in INS gene promoter	Regulation of insulin autoantigen expression increasing the risk of beta cells destruction.	(63, 77)
	SNPs in IL2RA gene	Regulation of the balance/homeostasis between effector and regulator T cells.	(64)
	SNPs in IFIH1 gene	Increased risk of T1DM and potentially of GDM.	(65)

GDM, gestational diabetes mellitus; DM1, diabetes mellitus type 1; GADA, glutamic acid decarboxylase autoantibodies; ICA, islet cell autoantibodies; IA-2A, insulinoma-associated protein 2 autoantibodies; ZnT8A, zinc transporter 8 autoantibodies; Treg, regulatory T cells; Th1/Th17, T cell subpopulations responsible for inflammatory response; IL-6, interleukin 6; IL-17, interleukin 17; TNF-alfa, tumor necrosis factor alpha; JAK/STAT, Janus kinase/signal transducer and activator of transcription; HLA-DR3, HLA-DR4, human leukocyte antigen DR3/DR4; CTLA4, cytotoxic T-lymphocyte antigen 4; PTPN22, protein tyrosine phosphatase non-receptor type 22; INS, insulin gene; IL2RA, interleukin 2 receptor alpha; IFIH1, interferon induced with helicase C domain 1.

## Diagnostics and clinical implications

Autoimmune gestational diabetes mellitus (autoimmune GDM) is a form of gestational diabetes in which glucose intolerance, first diagnosed during pregnancy, occurs in the presence of autoantibodies against pancreatic beta cells, suggesting immune-mediated damage to these cells and predisposing to the development of type 1 diabetes after delivery. Clinical characteristics of patients at risk of developing autoimmune GDM include: young age, low BMI, early initiation of insulin therapy and the presence of ketone bodies. The co-occurrence of two or more of these characteristics may indicate a need to perform an antibody titer test (28). The course of autoimmune GDM may resemble that of typical GDM, which makes clinical distinction of the two conditions difficult. However, autoimmune GDM more frequently progresses to T1DM after delivery, unlike typical GDM which disappears after gestation (16). There have been case studies where autoimmune GDM was manifested by ketoacidosis during pregnancy or puerperium, which is usually the first symptom of newly diagnosed T1DM (79, 80). Autoimmune GDM may require a more aggressive treatment of hyperglycemia, including earlier introduction of insulin therapy (81).

Untreated or poorly controlled diabetes in pregnancy, including the autoimmune GDM, has a significant impact on the developing fetus. Chronic maternal hyperglycemia causes excess glucose to pass through the placenta, which triggers hyperinsulinemia in the fetus. Since insulin has anabolic action, a classical complication is fetal macrosomia – excessive body mass and fatty tissue growth. Newborns of GDM mothers are at a significantly higher risk of being large for gestational age (LGA) and of macrosomia (birth weight >4000 g) compared to the general population (82). Macrosomia, in turn, is associated with complications during labor and delivery: it increases the risk of shoulder dystocia, perinatal injuries (clavicle fracture, brachial plexus injury) and perinatal hypoxia. Obstetric interventions are more frequently required, including vacuum extraction (VE), forceps or caesarean section, to safely deliver the baby. Importantly, appropriate GDM treatment (diet, insulin) significantly reduces the risk of macrosomia – with well-controlled glycemia most pregnancies result in the delivery of an infant with a normal or only slightly increased birth weight. In the context of GDM, it should be noted that in some analyses, the mere presence of autoantibodies in the mother did not increase the risk of LGA in the child, compared to other GDM cases (83). The mere presence of autoantibodies does not necessarily imply an increased risk for the fetus, thereby underscoring the heterogeneity of the phenotype. Autoimmune GDM should therefore not be regarded as a uniform clinical entity.

A second complication commonly found in the infants of GDM mothers is neonatal hypoglycemia. Congenital hyperinsulinism (HI or CHI) goes away shortly after birth, as the glucose supply from maternal blood stops once the umbilical cord is cut. It may result in an abrupt fall in the infant’s blood sugar level during the initial hours after birth. Babies of GDM mothers have an over tenfold risk of developing neonatal hypoglycemia – one study revealed that the prevalence of hypoglycemia in the neonates of diabetic mothers was approximately 11 times higher than in the babies of non-diabetic women (84). From the autoimmune GDM perspective, it is worth noting that in a multi-centre prospective cohort study the prevalence of neonatal hypoglycemia was significantly higher when autoantibodies were present in the mother (40% vs 12,5% of cases) (6). Perhaps, it was due to the fact that in this group of patients, insulin therapy and intense treatment were more frequently used, which triggered episodes of low glycemia in the infants.

Autoimmune GDM carries a higher risk of adverse outcomes in pregnancy, such as preeclampsia, premature labor and neonatal hypoglycemia, which requires intense monitoring and treatment. Gestational hypertension was found to occur significantly more often in autoimmune GDM (33% vs 1.7%, *p* < 0.001) (6). In the context of the course of pregnancy, the mechanism of hyperglycemia is less important than the degree of glycemic control. Regardless of the cause of GDM, effective treatment is crucial and with autoimmune mechanism involvement it may be even more difficult.

Current guidelines do not recommend routine testing for autoantibodies in every patient newly diagnosed with gestational diabetes, as autoimmune GDM accounts for a low percentage of the total number of cases and the impact of such testing on clinical management of the condition is uncertain. Nevertheless, in some instances immune diagnostic tools should be taken into consideration. If GDM is accompanied by atypical characteristics suggestive of T1DM–i.e. young age of the pregnant patient, slim stature, symptoms of polydipsia and polyuria prior to treatment initiation or exceptionally high blood sugar level despite the lack of severe obesity – a titer test for anti-islet antibodies (GAD, ICA, IA-2, potentially IAA) is recommended. Detection of autoantibodies in pregnancy may help identify patients who require intense monitoring after delivery (32).

The American Diabetes Association recommends that all women after GDM are referred for a follow-up OGTT about 6–12 weeks postpartum and that they undergo life-long screening for diabetes at least every three years (85). Given their increased risk of quickly progressing pancreatic islet destruction, patients with an autoimmune GDM component may require more intense glycemic control. The most important factors are the number, persistence, and dynamics of autoantibodies (6). Therefore, re-testing is recommended 6–12 weeks postpartum (28). Seroconversion or an increase in antibody titers indicate a increased risk of progression and require close diabetological follow-up (86).

Additionally, in recent years, there have been reports regarding potential implementation of immunomodulatory therapy to inhibit the autoimmune process leading to beta-cell destruction. Although immunomodulators are still in the experimental phase, new treatment options may become available to patients with autoimmune GDM in the future. Autoimmune gestational diabetes poses a diagnostic and therapeutic challenge. In recent years, it has been suggested that this subgroup of patients should be identified more effectively already in pregnancy, which would facilitate a more individualized care. To date, there is a lack of consensus as to who should be tested for autoantibodies.

## Therapeutic strategies

The new therapeutic approach to autoimmune GDM involves mainly immunomodulation to prevent the development of T1DM. Patients with multiple autoantibodies and a positive family history of type 1 diabetes may potentially qualify for immunomodulatory therapy. However, at present, such interventions are restricted to clinical trials. Immunomodulatory therapies are still at the experimental stage. Data from animal studies and from non-pregnant humans suggest potential, but their safety during pregnancy remains unknown and carries potential risks. The prevention of T1DM development is an intensely investigated issue. The research involves immunotherapy, early risk group identification, immunomodulating protective vaccination, lifestyle modifications to support metabolic health and avoid triggering factors, such as viral infections and environmental toxins, which may initiate the autoimmune process.

Studies into IL-6 and IL-17 inhibitors suggest their potential efficacy in beta-cell protection. Given that Th17/Treg disequilibrium contributes to sustained activation of autoimmune responses, immunomodulatory strategies (i.e., IL-6R blockade) are being considered as potential means of restoring immune homeostasis. Although the available evidence is derived predominantly from preclinical studies, the underlying mechanism provides a strong theoretical rationale for this therapeutic direction. IL-6 inhibitors, such as tocilizumab and sarilumab, prevent IL-6 from binding to it receptor, which may reduce the inflammatory processes leading to beta-cell damage (87). Tocilizumab, administered so far in pregnant women in COVID-19 therapy for instance, has been linked to an increased risk of infection and immune disorders in newborns, which raises doubts regarding the safety of its use in this group of patients (88). As for IL-17, studies on rodents showed that once the inflammatory process within pancreatic islets had been initiated, the blocking of IL-17 pathway was effective in delaying and preventing T1DM onset (89). Despite promising findings, clinical research on humans is in its early stage and long-term consequences are still unknown.

Another study showed that teplizumab, which is an anti-CD3 antibody, may delay T1DM onset in high-risk group individuals. Findings suggest that immunotherapy with teplizumab may constitute a promising alternative in preventing the progression of GDM to T1DM (90). While teplizumab was approved by the FDA for T1DM prevention, its potential use in pregnancy requires further clinical research.

The identification of HLA-DR3, HLA-DR4 and other genes associated with T1DM, as well as screening for autoantibodies facilitate early identification of T1DM risk groups. However, mass testing is expensive and to date it is believed that it may lead to unnecessary interventions in patients who will not develop T1DM (91).

There are ongoing studies into antiviral vaccines, i.e. against enteroviruses potentially associated with T1DM development (92). Although there is evidence of a correlation between viral infections and risk of diabetes, the efficacy of such vaccines remains unclear and requires further investigation. Clinical research financed by such organizations as TrialNet evaluate the efficacy of various immunotherapies, including immunomodulator and anti-inflammatory drugs, in T1DM treatment and prevention. TrialNet is an international network of clinical studies financed by the National Institutes of Health (NIH) in the USA. The TrialNet program involves screening tests for autoantibodies associated with T1DM in risk group individuals as well as new immunomodulatory therapy studies (93). The aim of the DIPP program (Type 1 Diabetes Prediction and Prevention) conducted in Finland is early identification of children with a genetic predisposition to T1DM, i.e. those with HLA genotypes linked to this condition. The program involves screening and monitoring for autoantibodies as well as early interventions in case of T1DM risk detection (94). Further research is necessary in order to evaluate potential benefits and risks associated with the implementation of preventive interventions in women with GDM.

## From autoantibodies to beta-cell damage: an immunopathogenic cascade in GDM

**Figure 2:**
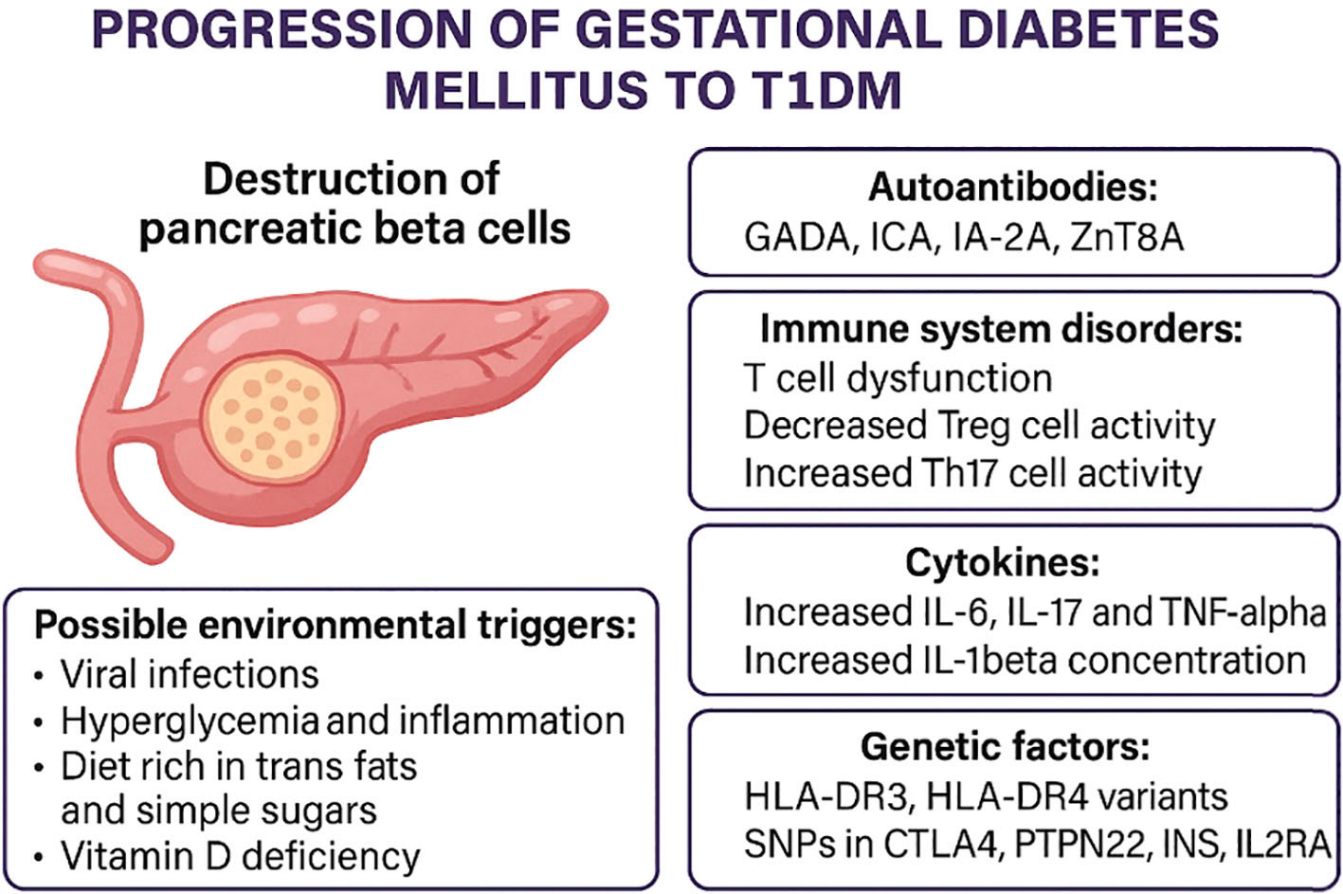
Autoimmune progression in gestational diabetes mellitus (GDM).

Autoantibodies directed against pancreatic beta-cell antigens represent the first indication of a potential autoimmune process in patients with GDM. Their presence reflects the activation of a specific immune response, which in some cases leads to further progression of autoimmunity. The subsequent stage of this process involves an imbalance between T-cell subpopulations particularly Th17 and Treg, as well as enhanced activity of pro-inflammatory cytokines (IL-6, TNF-α). These mechanisms, well described in the pathogenesis of T1DM, may serve as initiators of beta-cell damage in gestational diabetes with autoantibodies (**Figure 2**).

## Conclusion

In women with autoantibodies, chronic inflammation and genetic predispositions, there is a high risk of GDM progression to T1DM. It should be emphasized that not all studies indicate the justification for routine testing of autoantibodies in GDM [i.e (28)], which questions the rationale for their use as a screening test. Currently, their significance is limited to research studies and high-risk groups. Early identification of women with autoimmune GDM may improve pregnancy outcomes and prevent progression to chronic diabetes. Further research is required into the development of new diagnostic and therapeutic strategies for this group of patients.

A better classification of women with autoimmune GDM is essential. Screening tests to detect autoantibodies as well as evaluation of genetic risk factors may facilitate more precise monitoring and early implementation of therapeutic interventions. Such an approach may also reduce the risk of progression to a permanent form of autoimmune diabetes, particularly T1DM, after the pregnancy. It should be a priority of future research programs to find alternative methods of early diagnosis and modification of immune responses in women with autoimmune GDM through the inhibition of autoimmune response in a way that would minimize beta-cell destruction.

In the future, research should also focus on the identification of markers allowing early detection of autoimmune forms of GDM and on the development of personalized treatment strategies. A key uncertainty remains the role of autoantibodies in GDM, as their detection frequency is heterogeneous and the safety of potential immunomodulatory interventions raises concerns. An integration of immuno- and genetic diagnostics in the routine management of pregnant patients may significantly improve treatment efficacy and reduce long-term health complications.
